# CXCL13 predicts disease activity in early rheumatoid arthritis and could be an indicator of the therapeutic ‘window of opportunity’

**DOI:** 10.1186/s13075-014-0434-z

**Published:** 2014-09-24

**Authors:** Stinne Ravn Greisen, Karen Kræmmer Schelde, Tue Kruse Rasmussen, Tue Wenzel Kragstrup, Kristian Stengaard-Pedersen, Merete Lund Hetland, Kim Hørslev-Petersen, Peter Junker, Mikkel Østergaard, Bent Deleuran, Malene Hvid

**Affiliations:** Department of Biomedicine, Aarhus University, Building 1240, Wilhelm Meyers Allé 4, 8000, Aarhus, C, Denmark; Department of Rheumatology, Aarhus University Hospital, Norrebrogade 44, 8000 Aarhus, C, Denmark; Copenhagen Center for Arthritis Research, Center for Rheumatology and Spine Diseases, Glostrup Hospital, Nordre Ringvej 57, 2600 Copenhagen, Denmark; Department of Clinical Medicine, Faculty of Health and Medical Sciences, University of Copenhagen, Blegdamsvej 3, 2200 Copenhagen, Denmark; King Christian 10th Hospital for the Rheumatic Diseases and University of Southern Denmark, Campusvej 55, 5230 Odense, Denmark; Department of Rheumatology, Odense University Hospital, Sdr. Boulevard 29, 5000 Odense, C, Denmark; Department of Clinical Medicine, Aarhus University Hospital, Nørrebrogade 44, 8000 Aarhus, Denmark

## Abstract

**Introduction:**

A key phenomenon in rheumatoid arthritis is the formation of lymphoid follicles in the inflamed synovial membrane. C-X-C motif chemokine 13 (CXCL13) is central in this process as it attracts C-X-C chemokine receptor type 5 (CXCR5)-expressing B cells and T follicular helper cells to the follicle. We here examine the role of CXCL13 and its association with disease in patients with treatment-naïve early rheumatoid arthritis.

**Methods:**

Plasma samples from patients in the OPERA trial were examined for CXCL13 at treatment initiation and after 6 months of treatment with either methotrexate plus placebo (DMARD) (*n* = 37) or methotrexate plus adalimumab (DMARD + ADA) (*n* = 39). Treatment outcome was evaluated after 1 and 2 years. CXCL13 plasma levels in healthy volunteers (*n* = 38) were also examined.

**Results:**

Baseline CXCL13 plasma levels were increased in early rheumatoid arthritis patients in comparison with healthy volunteers. Also, plasma CXCL13 correlated positively with disease activity parameters; swollen joint count 28 (rho = 0.34) and 40 (rho = 0.39), visual analog score (rho = 0.38) and simplified disease activity index (rho = 0.25) (all *P* <0.05). CXCL13 levels decreased a significantly twofold more in the DMARD + ADA group than in the DMARD group. Baseline CXCL13 plasma levels in the DMARD group correlated inversely with disease activity parameters; disease activity score in 28 joints, four variables, C-reactive protein based (DAS28CRP) (rho = 0.58, *P* <0.05) at 12 months. High baseline CXCL13 was associated with remission (DAS28CRP less than 2.6) after 2 years.

**Conclusions:**

In treatment-naïve early rheumatoid arthritis patients, plasma CXCL13 levels were associated with joint inflammation. Furthermore, patients with high baseline plasma CXCL13 levels had an improved chance of remission after 2 years. We propose that high CXCL13 concentrations indicate recent onset of inflammation that may respond better to early aggressive treatment. Thus, high levels of CXCL13 could reflect the ‘the window of opportunity’ for optimal treatment effect.

**Trial registration:**

Clinicaltrial.gov NCT00660647. Registered 10 April 2008

## Introduction

Rheumatoid arthritis (RA) is a chronic autoimmune disease with joint inflammation and autoantibody production as key elements of its pathogenesis. The course of the disease is still difficult to predict. The encouraging results of early, intensive treatment of RA suggest the existence of a ‘window of opportunity’ during which effective therapy can induce long-lasting remission [1]. Unfortunately, it is not known when this ‘window of opportunity’ is open, and the search for informative biomarkers of early inflammation and triggers of memory development therefore becomes a pertinent issue in RA research.

T cells are present in elevated numbers in the synovial joints in RA where they form cellular infiltrates that resemble ectopic lymphoid aggregates with germinal center formation [2]. This suggests the presence of an ongoing antigen presentation and follicle formation in the synovium. The follicle is a well-organized structure, generated by follicular dendritic cells (FDCs), B cells, and follicular helper CD4 T (T_FH_) cells. Within the follicle, B cells are activated and matured into long-lived plasma cells, which secrete high-affinity antibodies [3]. The production of autoantibodies is central in RA [4], and the processes leading to follicle formation in the RA synovium are therefore of great interest. The central role of ongoing immune activation in RA development is further supported by the fact that CTLA4 treatment reduces disease activity [5].

The chemokine C-X-C motif chemokine 13 (CXCL13) is necessary for follicle formation and is constitutively expressed in secondary lymphoid tissue, primarily by FDCs [6]. Further, CXCL13 expression is upregulated by tumor necrosis factor alpha (TNFα) and by T cell receptor stimulation [7,8]. C-X-C chemokine receptor type 5 (CXCR5), the only known receptor for CXCL13, is expressed by naïve B cells and T_FH_ cells, and it controls the migration of these cells to the follicle [9]. The CXCL13-CXCR5 axis is critical to the generation of immunological memory based on long-lived plasma cells because the interaction between T_FH_ and B cells is necessary for the formation of plasma cells and autoantibody production [7,10]. Recently, CXCL13 has risen to be a possible new marker of disease and inflammation in RA. CXCL13 is reported upregulated in RA patients, and is suggested to be connected with both disease activity and rheumatoid factor [11,12].

In this study, we aim to investigate CXCL13’s association with markers of disease activity in patients with early RA, who participated in a double-blind randomized clinical trial of two different treatment regimes.

## Materials and methods

### Collection of patient samples and clinical data

A longitudinal set of plasma samples was obtained from a randomly selected subset of patients (n = 76, age = 55.4 (52 to 59), 72% women) who participated in the OPERA study (OPtimized treatment algorithm in Early Rheumatoid Arthritis). The trial was conducted in accordance with the Declaration of Helsinki and approved by the Danish Medical Agency (2612–3393), the Danish Data Protection Agency (2007-41-0072) and the Regional Ethics Committee (VEK-20070008). All patients gave written consent to participate in the study. The study design has been described in detail elsewhere [13]. Briefly, the patients were early treatment-naïve RA patients whose symptoms had lasted less than six months. Upon entry into this double-blind study, patients were randomized to conventional methotrexate (MTX) treatment plus placebo (disease-modifying anti-rheumatic drug (DMARD)) or MTX in combination with adalimumab (DMARD + ADA); both regimes were given in combination with intra-articular triamcinolone injections. If patients experienced a flare in disease, treatment was optimized. In relation to a change in treatment regime, the patients received intra-articular triamcinolone injections. Different treatment regimes are described in details in the original study [13]. In the present study, we used plasma samples obtained before the initiation of treatment (baseline) and after 6 months of treatment. At baseline, immunoglobulin M-rheumatoid factor (IgM-RF) and anti-citrullinated protein antibody (anti-CCP) were assessed. Disease activity was assessed each time plasma samples were collected using C-reactive protein (CRP), number of swollen (SJC 28 and 40) and tender joints (TJC 28 and 40), and physician’s global assessment of disease activity measured by a visual analog scale (VAS physician global), simplified disease activity index (SDAI), the disease activity score in 28 joints (DAS28CRP, four variables, CRP-based) and total Sharp Score (TSS). After the first year of treatment, adalimumab was discontinued and patients were continuously followed and treated for disease flare. DAS28CRP <2.6 was defined as remission. The patients’ clinical characteristics are presented in Table 1. Plasma samples were also collected from gender- and age-matched healthy volunteers (HVs) (n = 38, age median 54.8 (38 to 62), 67% women).

**Table 1:** **Patient characteristics**

	**Baseline**	**6 months**
**DAS28CRP**	5.7 (5.1-6.5)	2.1 (1.8-2.8)
**Swollen joint count (0-28)**	10 (7.0-17)	0 (0-0)
**Tender joint count (0-28)**	12 (7.0-18)	0 (0-1.3)
**Swollen joint count (0-40)**	13 (9.0-22)	0 (0-0)
**Tender joint count (0-40)**	17 (11-26)	0 (0-3.0)
**VAS doctor global (0-100 mm)**	56 (41-73)	2.0 (0-10)
**CRP (mg/ml)**	15 (7.0-42)	7.0 (7.0-7.0)
**SDAI (0.7-82)**	37 (29-47)	3.1 (0.86-7.0)
**IgM-RF (% positive)**	70.7%	-
**Anti-CCP (% positive)**	62.7%	-
**TSS (% positive)**	17.3%	-

Relevant disease markers at baseline and following 6 months of treatment. Data are expressed as median with interquartile range (IQR). Anti-CCP: anti-citrullinated protein antibody; CRP: C-reactive protein; DAS28CRP: disease activity in 28 joints, four variables, C-reactive protein based; IgM-RF: IgM rheumatic factor; SDAI: simple disease activity index; TSS: total Sharp score; VAS: visual analog scale.

### ELISA

Plasma CXCL13 levels were quantified according to the manufacturer’s instructions using a commercially available sandwich enzyme-linked immunosorbent assay (ELISA) kit (Quantikine human CXCL13/BCL/BCA-1, #DCX130 R&D systems, Minneapolis, MN, USA). All samples were diluted 1:2 in Calibrator Diluent RD6-41 supplemented with mouse and bovine IgG to ensure preaggregation of heterophilic antibodies. Samples were analyzed in duplicates, and the minimum detection limit (cutoff) was calculated as two standard deviations of the blanks. Values below the cutoff value were assigned the same value as the cutoff. The ELISA kit was validated as previously described by Kragstrup *et al*. [14].

### Statistics

Statistical analyses were performed using GraphPad Prism 5.0 for Mac (GraphPad Software, Inc., La Jolla, CA, USA). ELISA data were analyzed using the Mann-Whitney *U* test for nonpaired data and the Wilcoxon matched pairs test for paired data. Data are expressed as median with interquartile range (IQR). Nonparametric paired data were assessed for statistical correlation using Spearman’s rho. In all tests, the level of significance was a two-sided P value of less than 0.05.

## Results

### Plasma levels of CXCL13 in early RA

**Figure 1:**
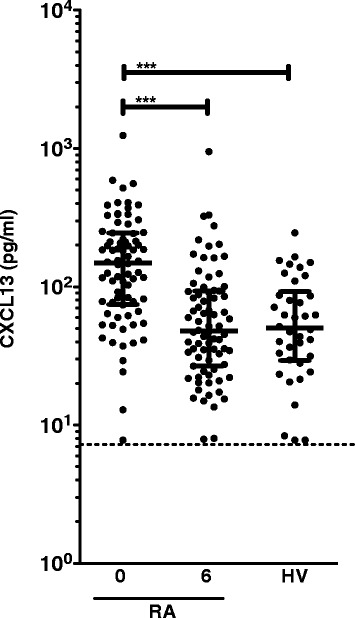
**Plasma levels of CXCL13 in early RA patients and healthy volunteers.** Levels of CXCL13 in plasma from early-stage RA patients (n = 76) and healthy volunteers (n = 38). Plasma CXCL13 levels were measured at treatment initiation (0) and after 6 months of treatment (6). Bars represent median with interquartile range. The cutoff level for detection was 7.8 pg/ml (dotted line). All values below the cutoff were assigned the cutoff value. Level of significance is indicated by asterisks (***: *P* <0.0001). CXCR13: C-X-C chemokine receptor type 13; RA: rheumatoid arthritis.

In patients with early RA plasma levels of CXCL13 at baseline were median (149.3 pg/ml (range 74.8 pg/ml to 245.0 pg/ml)). After 6 months of treatment plasma CXCL13 decreased threefold to 48.1 pg/ml (26.9 pg/ml to 93.0 pg/ml), P <0.001. CXCL13 levels at 6 months were similar to those observed in HVs (50.3 pg/ml (29.2 pg/ml to 92.7 pg/ml)) (Figure 1).

### Additional adalimumab treatment resulted in a greater degree of CXCL13 inhibition

**Figure 2:**
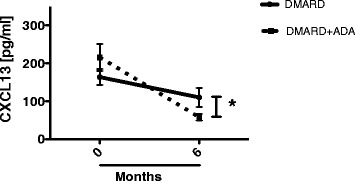
**Change in CXCL13 plasma levels in the two treatment groups.** Lines represent the median decrease in plasma CXCL13 levels from 0 to 6 months, in the DMARD + ADA (full line) and DMARD (dotted line) groups. *Indicates a statistically significant difference between the changes in the two groups (*P* <0.05). ADA: adalimumab; CXCR13: C-X-C chemokine receptor type 13; DMARD: disease-modifying anti-rheumatic drug.

We assessed plasma CXCL13 levels in the two treatment groups separately. At baseline, the plasma CXCL13 levels in the DMARD + ADA group were not significantly different from the plasma CXCL13 levels in the DMARD group. Although the plasma CXCL13 levels in both groups after 6 months of treatment did not differ from those of HVs, they were reduced 4.2-fold in the DMARD + ADA group, but only 1.9-fold in the DMARD group (*P* <0.05) (Figure 2).

### CXCL13 was associated with core disease parameters

We analyzed the association between CXCL13 levels and clinical disease activity parameters at baseline and after 12 months of treatment. At baseline, CXCL13 plasma levels correlated positively with SJC28 (rho = 0.336, *P* = 0.003) and SJC40 (rho = 0.392, *P* = 0.001) (Table 2). It also correlated with VAS physician global (rho = 0.378, *P* = 0.001) and SDAI (rho = 0.254, *P* = 0.028) (Table 2). CXCL13 level at baseline showed no association with clinical disease activity parameters after 12 months of treatment (Table 2). At six months, we neither observed associations between plasma CXCL13 levels and the disease activity parameters, nor did we observe correlation between CXCL13 and rheumatoid factor (data not shown). We did not observe correlation with TSS at any time point.

**Table 2:** **Correlations of CXCL13 plasma levels with disease activity parameters at baseline and following 6 months of treatment**

**Time**	**Disease marker**	**0 months rho (** ***P*** **)**	**6 months rho (** ***P*** **)**
**Baseline**			
	IgM-RF	−0.112 (0.34)	0.073 (0.54)
	Anti-CCP	0.021 (0.86)	0.099 (0.31)
	TSS	−0.011 (0.92)	0.059 (0.62)
	**Swollen joint count 28**	**0.336 (0.003)**	0.110 (0.35)
	Tender joint count 28	0.166 (0.16)	0.001 (0.99)
	**Swollen joint count 40**	**0.392 (0.001)**	0.177 (0.13)
	Tender joint count 40	0.162 (0.17)	0.007 (0.95)
	**VAS doctor global**	**0.378 (0.001)**	0.103 (0.38)
	CRP	0.094 (0.42)	0.145 (0.21)
	DAS28CRP	0.205 (0.078)	0.089 (0.45)
	**SDAI**	**0.254 (0.028)**	0.091 (0.44)
**12 months**			
	Swollen joint count 28	0.131 (0.27)	−0.077 (0.51)
	Tender joint count 28	0.195 (0.096)	−0.012 (0.92)
	Swollen joint count 40	0.162 (0.17)	−0.085 (0.47)
	Tender joint count 40	0.219 (0.060)	0.045 (0.71)
	VAS doctor	0.006 (0.96)	−0.037 (0.76)
	CRP	−0.047 (0.69)	−0.124 (0.30)
	DAS28CRP	0.059 (0.62)	0.012 (0.92)
	SDAI	0.009 (0.94)	0.047 (0.67)

Correlations of clinical data with the plasma level of CXCL13 measured at 0 months and after 6 months of treatment, in the OPERA trial. Correlations are presented as Spearman’s rho (*P* value). *P* values lower that 0.05 are considered statistically significant (indicated by **bold**). Statistically significant correlations between plasma CXCL13 level and disease parameters were observed at baseline, but not following treatment. Anti-CCP: anti-citrullinated protein antibody; CXCR13: C-X-C chemokine receptor type 13; CRP: C-reactive protein; DAS28CRP: disease activity in 28 joints, four variables, C-reactive protein based; IgM-RF: IgM rheumatic factor; OPERA: OPtimized treatment algorithm in Early Rheumatoid Arthritis; SDAI: simple disease activity index; TSS: total Sharp score; VAS: visual analog scale.

### High baseline CXCL13 in the DMARD-treated group was associated with low SDAI and VAS score at one year

**Figure 3:**
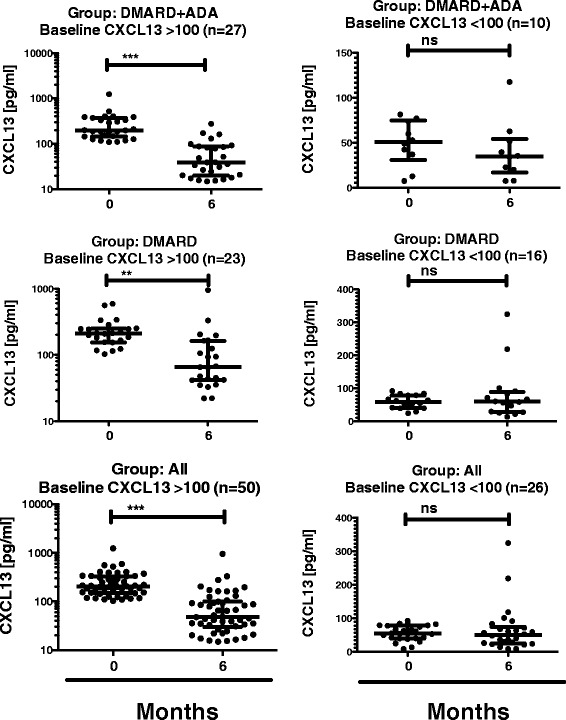
**Plasma CXCL13 at 0 and 6 months, in patients with high- and low-level CXCL13 in the treatment groups.** Plasma levels of CXCL13 at 0 and 6 months in the DMARD group, the DMARD + ADA group and all patients, subdivided into ‘CXCL13-*high’* and ‘CXCL13-*low’* according to baseline level of CXCL13 ≥100 vs. <100. ***Indicates *P* <0.001, **: *P* <0.01, and ns: *P* >0.05. ADA: adalimumab; CXCR13: C-X-C chemokine receptor type 13; DMARD: disease-modifying anti-rheumatic drug.

Since CXCL13 plasma levels varied widely at baseline, we aimed to identify subgroups within the cohort. We divided the patients into two groups according to their CXCL13 plasma levels at baseline with CXCL13-*high* >100 pg/ml and CXCL13-*low* <100 pg/ml as described by Rosengren *et al.* [11]. Treatment induced no significant change in CXCL13 plasma levels in the CXCL13-*low* group, but a significant decrease was seen in the CXCL13-*high* group (Figure 3). Scrutiny of the CXCL13-*high* DMARD group revealed that the baseline CXCL13 level had a significant, negative correlation with a range of variables reflecting disease activity at 12 months: VAS doctor (rho = −0.598, *P* = 0.003), CRP (rho = −0.504, *P* = 0.02), DAS28CRP (rho = −0.582, *P* = 0.006), and SDAI (rho = −0.589, *P* = 0.006). In the DMARD + ADA group, however, no similar correlations with disease markers were observed. We did not observe any difference in baseline CRP between the CXCL13-*high* and -*low* group (data not shown).

### Sustained remission after 2 years was associated with high baseline CXCL13

**Figure 4:**
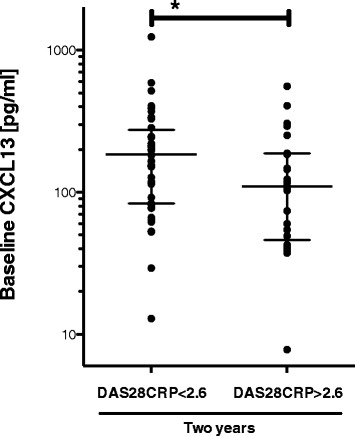
**Baseline CXCL13 stratified by clinical disease activity score (+/−DAS28-remission) after 2 years of therapy.** Both treatment groups are considered together. Bars represent median with IQR. *Indicates statistically significant difference (*P* = 0.03). CXCR13: C-X-C chemokine receptor type 13; DAS28: disease activity score in 28 joints; IQR: interquartile range.

We examined patients who were in remission (DAS28CRP <2.6) at the 2-year follow-up (n = 48). These patients had a high baseline CXCL13 level (184.2 pg/ml (83.46 to 275.2)), whereas patients in non-remission (DAS28CRP >2.6, (n = 25)) had a lower baseline CXCL13 level (110.3 pg/ml (45.95-187.6), *P* = 0.014) (Figure 4). When analyzed per randomization group, we obtained similar results for the DMARD + ADA group, and additionally here, 89% of patients in remission were in the CXCL13-*high* baseline group. In the DMARD group, 59% of patients in remission after two years were in the CXCL13-*high* baseline group (data not shown).

We repeated the same analysis evaluating CRP <8 mg/L at 2-year follow-up. Here, we observed no difference in CXCL13 plasma level at baseline.

**Figure 5:**
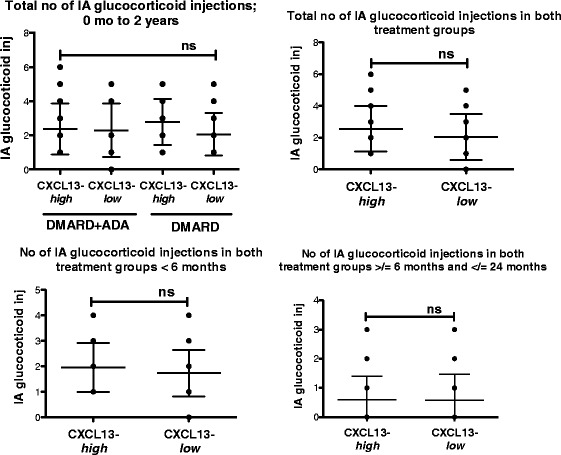
**Number of intra-articular triamcinolone injections in patients from the CXCL13-** ***high*** **and -** ***low*** **group between baseline and two years.** Aligned dot-plot of the number of intra-articular injections is presented as total number of injection between baseline and two years. CXCL13-*high* DMARD + ADA (n = 27) and DMARD (n = 23), CXCL13-*low* DMARD + ADA (n = 10) and DMARD (n = 16). Further, the number of intra-articular injections is stratified into number of injections before six months and between six months and 2 years (mean with SD). ADA: adalimumab; CXCR13: C-X-C chemokine receptor type 13; DMARD: disease-modifying anti-rheumatic drug; SD: standard deviation.

The OPERA is a treat-to-target protocol, where treatment is optimized by intra-articular triamcinolon injections following a strict optimization protocol [13]. To exclude that the CXCL13-*high* group received a more aggressive treatment than the CXCL13-*low* group we used the number of intra-articular injections between baseline and two years, and we investigated if patients had been receiving additional DMARDs than MTX (hydroxychloroquine and/or sulphasalzine). Patients in the CXCL13-*high* baseline group did not differ from patients in the CXCL13-*low* group in regard to change in treatment regimes (Figure 5 and Table 3).

**Table 3:** **Additional treatment in CXCL13-** ***high*** **and CXCL13-** ***low*** **group**

	**DMARD + ADA**		**DMARD**	
	**CXCL13-** ***high***	**CXCL13-** ***low***	**CXCL13-** ***high***	**CXCL13-** ***low***
Additional treatment	6/27, 22.2%	4/10, 40%	9/23, 39,1%	6/16, 37,5%

Number of patients in the CXCL13-*high* and -*low* group treated with additional DMARDs than MTX. If sulphasalazine, hydroxychloroquine or both has been added to the treatment during the 2-year follow-up patients will be considered to be receiving additional treatment. x/y represents the number of patients receiving additional treatment/number of patients in the group. ADA: adalimumab; CXCR13: C-X-C chemokine receptor type 13; DMARD: disease-modifying anti-rheumatic drug.

## Discussion

In this study, we further investigated the role of CXCL13 in RA. We measured high CXCL13 plasma levels in early DMARD-naïve RA patients. Six months of anti-rheumatic treatment reduced plasma CXCL13 to levels observed in healthy volunteers. We also showed that baseline CXCL13 strongly correlated with SDAI, VAS and joint involvement at treatment initiation. These findings contribute to establishing a role for CXCL13 as a potential marker of inflammation in early RA. Our findings are in line with earlier published results on CXCL13 [11,15,16], but our study provides new knowledge suggesting CXCL13 as a marker of joint involvement in early RA.

CXCL13 is a pivotal chemokine in establishing an adaptive immune response. It attracts B cells in the secondary lymphoid tissue, which facilitates the generation of antibodies and local inflammation [6,7]. The observed associations with joint involvement contribute to establishing activity within the lymphoid follicle in early RA as an important mechanism in the progression of RA. Because CXCL13 is produced by synovial cells, CXCL13 could serve as a marker that reflects local activity and inflammation [8]. CXCL13 was not associated with CRP or DAS28CRP. Rioja *et al*. [17] describes high CXCL13 and DAS28 levels in patients with active vs. inactive RA. In line with these findings, we observed that CXCL13 levels are high in untreated early RA patients (active RA), as is DAS28CRP and CRP. Treatment of early RA reduces disease activity, and thereby also DAS28CRP as well as CXCL13. Thus, although not associated with CRP, CXCL13 remains a potential marker of disease activity in early RA patients.

In the DMARD treated CXCL13-*high* group, the baseline CXCL13 levels correlated inversely with disease activity markers at 12 months. *A priori*, one would not expect high levels of CXCL13 to correlate inversely with disease parameters. Rosengren *et al*. [11] described plasma CXCL13 levels to decrease in accordance with disease activity, indicating CXCL13 and disease parameters to be positively correlated. However, Rosengren *et al*. examined patients with established RA. Bugatti *et al.* [15] find fewer patients in clinical remission after one year of treatment, if baseline levels of CXCL13 were high. In line with Bugatti *et al*.’s study, Meeuwsisse *et al*. [16] show that high CXCL13 is associated with increased radiographic destruction. We do not find any association with radiographic progression. Our results are of course controversial in comparison with both Meeuwisse *et al*. and Bugatti *et al*.’s findings. Though the average disease duration in our cohort is only 3 months, where disease duration in Bugatti’s cohort is 1 year and 2 years in Meeuwisse’ cohort. We suggest this difference is of major importance, as these very early RA patients comprise a more uniform cohort, because spread in disease increases significantly over time. Our different findings can be explained by the fact that our patients are still in the earliest phases of disease initiation. Also supporting the difference in the patient cohorts is that 67% of patients in Bugatti *et al*.’s article reached low disease activity after one year, whereas this percentage was 76 to 80% in the OPERA cohort. Again supporting a difference is when patients are treated aggressively and as early as after just 3 months of disease. Jones *et al*. [12] recently showed, that CXCL13 is associated with rheumatoid factor in RA patients, supporting its importance in antibody production. In our cohort of patients with very early RA, and we did not observe CXCL13 to be associated with rheumatoid factor. Thus, we propose that a high, plasma CXCL13 level in treatment-naïve early RA is a possible indicator of newly developed and reversible inflammation. It is likely that these very early RA patients have neither established a full memory response, nor fully developed a lymphoid follicle antigen response at this earliest stage of disease. This would imply that the memory process to some degree could be halted, possibly by aggressive treatment regimes. In the DMARD + ADA treated CXCL13-*high* group we do not see this inverse correlation with disease markers. Several studies on TNF^−/−^ mice elucidate the importance of TNF receptors such as TNF-R1 in fully establishing an immune response [18-20]. Thus TNF is required for differentiation of follicular dendritic cells and an antibody response. This could explain the lack of associations in the DMARD + ADA treated group and reflect the difference in treatment response between the two groups. Thus, the DMARD + ADA-treated patients had decreased disease activity after 12 months of treatment compared with the DMARD-treated patients [13]. This supports the hypothesis that adding adalimumab to the treatment regime impairs the development of disease progression and possibly also immunologic memory, while disease progression in the DMARD group is ongoing. We also showed that sustained remission (measured by DAS28CRP <2.6) at 2 years of follow-up, was associated with higher baseline CXCL13. This finding could further support that high baseline CXCL13 may be an indicator of recent-onset and active disease, and that an ‘open window’ for successful treatment does exist when the disease is in its earliest phase. We analyzed if patients with high CXCL13 simply were treated more aggressively, and therefore achieved sustained remission. This was not the case, as evaluated by number of intra-articular steroid injections and addition of hydroxychloroquine and/or sulphasalazine. When we repeated the above analysis, using CRP with a cut of 8 mg/L as a definition of remission, no difference in baseline CXCL13 was observed. This supports the theory that CXCL13 especially reflects joint involvement, and is not just connected to CRP. Based on these very early RA patients from the OPERA cohort, we propose that an initial high level of CXCL13 could be a potential indicator that the patients are more treatment-responsive and thereby within the so-called ‘window of opportunity’. Adding adalimumab to the treatment regime seems to further improve the chance for remission after two years, especially with high baseline CXCL13. Our findings may therefore also contribute to the explanation of the disease-modifying effects of early aggressive treatment.

## Conclusions

Our study suggests that plasma CXCL13 is a marker of early inflammation in general and especially of joint involvement in early RA. Early RA patients with high baseline CXCL13 levels could form a particular patient group whose disease is still very responsive to treatment. This responsiveness could indicate that patients are in the earliest disease stage and within the ‘window of opportunity’ where they probably respond better to early aggressive treatment than patients whose disease has progressed.

## Abbreviations

ADA: adalimumab
anti-CCP: anti-citrullinated protein antibody
CRP: C-reactive protein
CXCR5: C-X-C chemokine receptor type 5
CXCL13: C-X-C motif chemokine 13
DAS28CRP: disease activity in 28 joints, four variables, C-reactive protein based
DMARDs: disease-modifying anti-rheumatic drugs
ELISA: enzyme-linked immunosorbent assay
FDCs: follicular dendritic cells
HV: healthy volunteers
IgM-RF: IgM rheumatic factor
IQR: interquartile range
MTX: methotroxate
OPERA: OPtimized treatment algorithm in Early Rheumatoid Arthritis
RA: rheumatoid arthritis
SDAI: simple disease activity index
SJC: swollen joint count
T_FH_: follicular T helper cells
TJC: tender joint count
TNFα: tumor necrosis factor α
TSS: total Sharp score
VAS: visual analog scale
